# Ovarian cancer is detectable from peripheral blood using machine learning over T-cell receptor repertoires

**DOI:** 10.1093/bib/bbae075

**Published:** 2024-03-13

**Authors:** Miriam Zuckerbrot-Schuldenfrei, Sarit Aviel-Ronen, Alona Zilberberg, Sol Efroni

**Affiliations:** The Mina & Everard Goodman Faculty of Life Sciences, Bar-Ilan University, Ramat-Gan 5290002, Israel; Adelson School of Medicine, Ariel University, Ariel 40700, Israel and Sheba Medical Center, Tel-Hashomer, Ramat Gan 526200, Israel; The Mina & Everard Goodman Faculty of Life Sciences, Bar-Ilan University, Ramat-Gan 5290002, Israel; The Mina & Everard Goodman Faculty of Life Sciences, Bar-Ilan University, Ramat-Gan 5290002, Israel

**Keywords:** T-cell receptor repertoire, ovarian cancer, biomarkers, machine learning

## Abstract

The extraordinary diversity of T cells and B cells is critical for body maintenance. This diversity has an important role in protecting against tumor formation. In humans, the T-cell receptor (TCR) repertoire is generated through a striking stochastic process called V(D)J recombination, in which different gene segments are assembled and modified, leading to extensive variety. In ovarian cancer (OC), an unfortunate 80% of cases are detected late, leading to poor survival outcomes. However, when detected early, approximately 94% of patients live longer than 5 years after diagnosis. Thus, early detection is critical for patient survival. To determine whether the TCR repertoire obtained from peripheral blood is associated with tumor status, we collected blood samples from 85 women with or without OC and obtained TCR information. We then used machine learning to learn the characteristics of samples and to finally predict, over a set of unseen samples, whether the person is with or without OC. We successfully stratified the two groups, thereby associating the peripheral blood TCR repertoire with the formation of OC tumors. A careful study of the origin of the set of T cells most informative for the signature indicated the involvement of a specific invariant natural killer T (iNKT) clone and a specific mucosal-associated invariant T (MAIT) clone. Our findings here support the proposition that tumor-relevant signal is maintained by the immune system and is coded in the T-cell repertoire available in peripheral blood. It is also possible that the immune system detects tumors early enough for repertoire technologies to inform us near the beginning of tumor formation. Although such detection is made by the immune system, we might be able to identify it, using repertoire data from peripheral blood, to offer a pragmatic way to search for early signs of cancer with minimal patient burden, possibly with enhanced sensitivity.

## INTRODUCTION

Ovarian cancer (OC) causes more deaths than any other cancer of the female reproductive system [1]. The disease is often diagnosed late [2] and is composed of several subtypes with distinct biological and molecular properties [3] (even within the same histological subtype [4]), with an inconsistency in availability of and access to treatment [5]. Outcomes in OC depend on timely diagnosis and access to appropriate surgery and systemic therapy [6, 7]. OC is often diagnosed at stages III and IV, where the 5-year survival rate is only 17–39% [8].

This diagnostic delay results from a lack of effective screening options at an early stage and a lack of early, specific, warning signs or symptoms [9]. To date, population screening in OC has not been effective or validated. It is estimated that early detection of OC could reduce mortality by 10–30% [9].

The collection of all antibody and T-cell immune receptors within an individual is often referred to as the adaptive immune repertoire [10], and it represents both the ongoing and past immune status of an individual. In a healthy adult, 1 ml of peripheral blood repertoire contains data from approximately 50 000 to 1 500 000 B cells and 600 000 to 3 000 000 T cells [10]. Both the types and sizes of B- or T-cell clones act as signal for diagnostic purposes. Modifications in size indicate regulation in the form of an increased (or reduced) frequency of cells expressing specific receptors. See further discussion of the generality of the approach in Aranout *et al*. [10] and specific to cancer in Joshi *et al*. [11]. Such clone identities and clone sizes can then be quantified using adaptive immune receptor repertoire sequencing (AIRR-seq [12]) or repertoire sequencing (Rep-seq [13]). It is assumed that antigen encounter, clonal expansion and diversification result in a personalized record of a person’s immune status across vaccination, infection, autoimmunity, transplant rejection, transfusion reactions and cancer. Rep-seq makes it possible to read this record [10]. The specific sequencing of TCRs, TCR-seq, involves the use of high-throughput sequencing platforms to record large numbers of short DNA/RNA sequences [14], which enables the quantification of T-cell diversity at unprecedented resolution. A major key region that is covered by these methods is complementarity determining region 3 (CDR3). A potential application of this methodology is leveraging a TCR or a composition of a group of TCRs as a biomarker to predict clinical statuses such as diagnosis or prognosis. For example, when the tumor status is unknown, the possibility of obtaining information about the tumor status from peripheral blood is of special interest. To do that, the detailed characterization of the TCR profile in peripheral blood mononuclear cells (PBMCs) is essential [15].

## METHODS

We collected a total of 85 samples through collaboration with the Sheba Hospital Biobank and Tel Aviv Medical Center Biobank: 34 blood samples from newly diagnosed OC patients prior to any treatment (collected during surgery) with specific subtypes detailed in the accompanying report (Supplementary Table 1) and 51 blood samples from healthy female donors. Five milliliters of blood were collected from each sample. Whole blood was collected into ethylenediaminetetraacetic acid-coated tubes to allow the purification of PBMCs and serum fractions. The cell pellets were frozen in 1 ml aliquots with 10% dimethyl sulfoxide/90% fetal calf serum (FCS) and stored at –80°C until shipped to Bar-Ilan University. Each vial comprises approximately 1–1.5 × 10^6^ cells/ml. PBMC samples were thawed, and their total RNA content was extracted using the RNeasy mini kit.

The obtained RNA products were evaluated for their integrity value (RIN) to standardize quality. Next, RNA concentrations were determined by qubit, which provides an accurate measurement of the quantitation of low-abundance RNA samples. A fixed total RNA concentration of 200 ng from each sample was subjected to the SMARTer Human TCR a/b Profiling Kit V2 (Takara Bio). This kit enables the analysis of TCR repertoires from bulk RNA samples. It generates Illumina-compatible sequencing libraries.

The TCR sequencing library is then size-selected and purified using AMPure XP beads. The generated libraries were measured for their DNA concentration by qubit and assessed for their sizes using TapeStation. This allows the pool together of 24 libraries on each single flow cell, preserving an equal representation of each single library in the final pool.

Sequencing was performed on an Illumina sequencer using the 300-cycle NextSeq 500/550 Mid Output Kit v2.5 and using paired-end sequencing with 2 × 150 base pair reads. That specific format enables us to capture the CDR3 domain of each TCR α/β transcript.

The output files (FASTQ) from the Illumina sequencer are analyzed using the Cogent NGS Immune Profiler Software. This software is an analysis pipeline tool that enables high-quality TCR profiling analysis by (i) collapsing unique molecular identifiers (UMIs) into their original number of transcripts using MiGEC [16] and (ii) aligning sequences using MiXCR [17] to extract the CDR3 sequences and their abundance in each sample. This results in a CDR3 composition per sample and a report with QC analysis and mapping statistics.

Part of the analysis used Immunarch [18], an open-source tool (an R package) designed to analyze immune repertoire data, T-cell receptor (TCR) and B-cell receptor (BCR) repertoires. With this tool, we perform basic statistics on the data from a quality control perspective. The different metrics included in the analysis provide important information about the dynamics of the TCR repertoire.

Maintaining the uniformity of samples is important for preventing bias. A commonly used strategy to generate comparable data involves subsampling. Here, we used the *repSample* function from the Immunarch [18] package with a downsampling method that downsamples repertoires to the number of clones (i.e. reads/UMIs) specified, usually to the number of clones in the smallest repertoire. It chooses clones from the input repertoires without any probabilistic simulation. During the downsampling process, samples of two healthy donors (HDs) were excluded due to their extremely small repertoires.

We utilized three databases for feature filtering: McPAS [19], VDJdb [20] and Trust4 TCGA [21]. The datasets were mined to retain unique rows and subsequently grouped by CDR3 sequence.

We built our classification model using ATOM [22], an open-source machine learning package for Python. ATOM also includes fast implementations of hyperparameter tuning methods.

We worked through multiple feature selection estimators with the ‘Select From Model’ (SFM) [23] algorithm and the ‘Sequential Feature Selection’ (SFS) [23] algorithm: linear discriminant analysis (LDA), XGBoost (XGB), LightGBM (LGB) and linear regression.

Not surprisingly, the high correlation between features led to different sets selected by each of the feature selection approaches. For each estimator, we tried eight different models, totaling 32 models. Optimal hyperparameters for each model were selected using Bayesian optimization (BO), with 10 starting points, for 40 iterations. Our motivation to use BO comes from its universal use in machine learning. The richness of the set of parameters rules out any possibility of a manual search or a random search. The exhaustiveness of grid search also prevents us from working with this method. In contrast, BO makes informed choices regarding which configurations to assess next by drawing from the results of prior evaluations, therefore working in a more efficient way. While it may have been possible to perform exhaustive searches when the number of features reduces to only a few (see below), the performances are at very high levels by then and do not seem to be restrained by the optimization method itself. The reason for the specific choices of starting points and iterations has been the ongoing experience with the runs, in which we noticed the number of starting points beyond 10 has very little influence over performance. Similarly, 40 iterations have presented themselves as a semi-optimal value.

During every step of the BO, the training set (as it was before any transformation) was split into a sub-train set and a validation set. This sub-train set is used for training the model, while the validation set is used to evaluate the selected model and hyperparameters in every call. Once the best combination of hyperparameters is found, the model is fitted on the entire training set. After that, the model is evaluated on the test set with a bootstrap algorithm (*n* = 5). This algorithm generates *n* new datasets by randomly selecting samples from the training set with replacement, ensuring that each bootstrapped dataset matches the size of the original training set. The model is then assessed on the test set. This approach yields a more resilient performance score estimation for the model, as it is derived from the average of *n* individual scores. It is worth noting that the same sets are utilized consistently across all models.

The best-performing model was selected based on the area under the receiver operating characteristic curve (AUC).

The execution time for fitting the model is 0.012 s. The model ran on an Intel(R) Xeon(R) CPU E5-2650 v3 @ 2.30GHz machine with 198 GB RAM.

All the codes used are available on GitHub at https://github.com/Miriam-Zu/Ovarian.

## RESULTS

**Figure 1:**
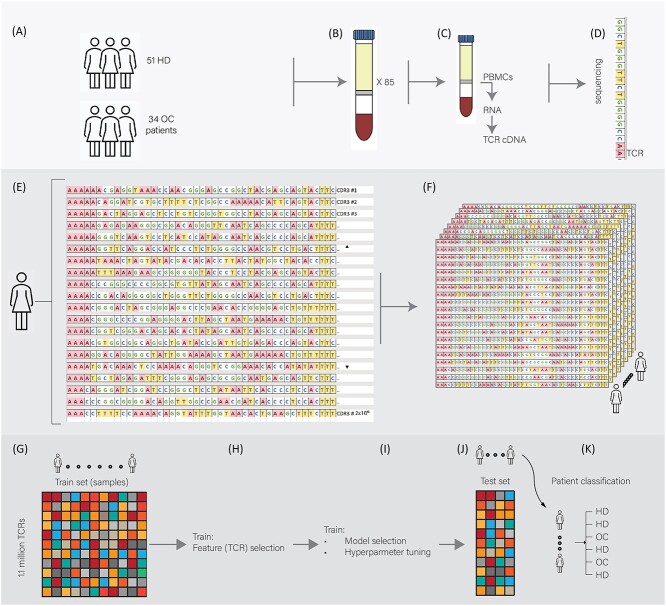
A visual representation of the outline for sample collection, sample processing and machine learning from the produced data. To produce these data, we (**A**) collected samples from 85 women with (34) or without (51) OC (see Methods for details). All samples from OC patients were collected prior to any treatment. After collection (**B**), we isolated PBMCs, produced RNA and built TCR-seq libraries (**C**), which were subjected to high-throughput sequencing (**D**). This workflow culminated in a TCR repertoire per individual (**E**), in which every patient is described by hundreds of thousands of clones and their abundance, leading to the description of the study population through their TCR repertoire (**F**). We represent these data using one large table (**G**), in which every row represents a clone (1.1 million clones) and every column represents the abundance of that clone in a specific patient (85 columns). We then train machine learning algorithms on these data and perform feature selection (**H**), eventually selecting a model (**I**) and tuning its hyperparameters. Finally, the model is tested on a set of patients previously held out from the training (**J**), classifying them as with or without OC (**K**).

Identifying associations between the TCR repertoire obtained from peripheral blood and tumor status is done using blood samples from OC patients and from HDs. Data from these samples, followed by machine learning, facilitated a repertoire-based model to classify previously unseen samples to their correct clinical status. That is, the model can determine whether a blood sample came from an OC patient or from an HD (Figure 1).

Learning and evaluation have been done in the manner detailed in Methods and reported in Supplementary Table 2 as a Data, Optimization, Model and Evaluation (DOME) [24] report. All reported results refer to the data after the required subsampling (see Methods).

### Quantification and assessment of clonotypic measures

Unique clonotypes were initially quantified across all samples (Supplementary Figure 1A). These number showed no significant differences between OC and HD in the quantities of unique sequences, in sequence overlap (Supplementary Figure 1B), in rare clonotypes (Supplementary Figure 1C) and in the most abundant clonotypes (Supplementary Figure 1D). We then measured Gini diversity, Gini–Simpson diversity and true diversity in all samples (Supplementary Figure 1E–G) and found no significant differences between the two groups (*P*-value < 0.13, 0.39 and 0.13, respectively). Interestingly, inverse Simpson index showed a significant difference (Supplementary Figure 1H, *P* < 0.048). However, this difference is not strong enough to provide sample classification.

### Supervised machine learning and feature selection for accurate classification

Straightforward statistical metrics were not able to discriminate OC samples from HD samples. Therefore, we used supervised machine learning for the task. For the learning, we use the following minimal representation: the dataset is composed of a data table with 83 columns (two samples were eliminated, see Methods) and 1 141 882 rows. Each column represents a sample (49 HD samples and 34 OC samples), and each row represents a TCR sequence. The value in each cell (row, column) is the frequency of a specific TCR clone in that specific sample. Training was performed on a randomly selected subsample (that is, randomly selected columns, see DOME model). Testing was performed on previously unseen samples (20).

**Figure 2:**
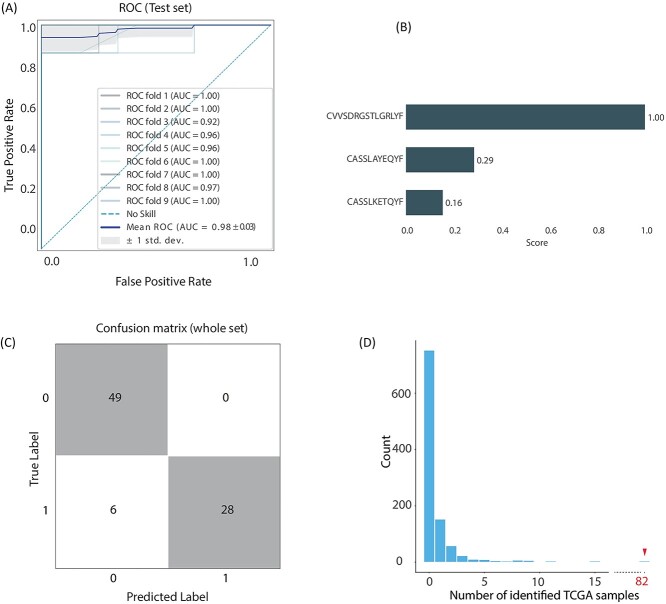
Results of top–down classification and feature selection. (**A**) A receiver operating curve representing multiple data splits shows robustness of the algorithm and an average receiver operating characteristic (ROC) curve in a darker shade. Average AUC value is 0.98. (**B**) As explained in the text, the final model was able to use three features (three clones) to achieve optimal results. The CDR3 sequences of these three clones are reported in the panel. Interestingly, the first clone belongs to an iNKT cell. (**C**) A confusion matrix showing performance over entire dataset. (**D**) To test the significance of the three clones presented in (B), we repeatedly, randomly, selected three TCRs and searched for them in the TRUST4 TCGA dataset. This was repeated 1000 times, with the number of appearances of at least one of the three TCRs recorded. As the panel indicates, the three TCRs we selected for the GBM model appear in significantly larger frequency.

We used two distinct methodologies for feature selection. Let us call these methodologies (i) top–down and (ii) top clones. The top–down methodology works by filtering TCRs for inclusion in the model based on sharing. That is, we included only shared clones. Specifically, our analysis began with the 600 top common clones within our dataset. Then, using SFM, we reduced the number of features (clones) to 10. The SFM procedure provided the best performance when we used gradient boosting (GBM) as a model with a feature set of size 10, which we selected using the light gradient boosting (LGB) estimator. The AUC for the test set was an outstanding 0.95. Feature ranking shows the non-uniform contribution of the features to the classification and indicated that not all 10 features in the set are necessary required for success in classification. Therefore, we continued to an additional feature selection step, to reduce the number of features from 10 to 3 using backward-SFS with a GBM estimator, followed by retraining of the model. Results show that this new model, which uses only three clones, is able to reach an average AUC on multiple splits of the data of 0.98. Feature ranking is shown in Figure 2B. Figure 2C illustrates the classification outcomes for the entire dataset (train + test).

The top clones methodology builds on our previous experience with ‘omnipresent’ clones, that is, clones that appear in large number of dissociated samples, observed by multiple research groups under multiple different conditions. Our approach to identify these clones is by using their prevalence in public databases. Specifically, we are using the databases VDJdb [20] and McPAS [19]. To these, we add the TCGA dataset obtained by the TRUST4 group [21], created through the mining of RNA-Seq files using TRUST4 [25] tool. Selection is performed by first obtaining the 10 most prevalent CDR3 sequences each database. Interestingly, five of the sequences overlapped, leaving us with a combined total of 25 sequences. Sixteen of these 25 sequences appeared in our (84 samples) dataset. With these 16 sequences at hand, we again implemented features selection algorithms as before, starting with backward-SFS algorithm, reducing the number to eight distinctive clones for further analysis. See Methods for details on (i) estimators, (ii) hyperparameter tuning using BO, (iii) training, (iv) validation and (v) testing.

**Figure 3:**
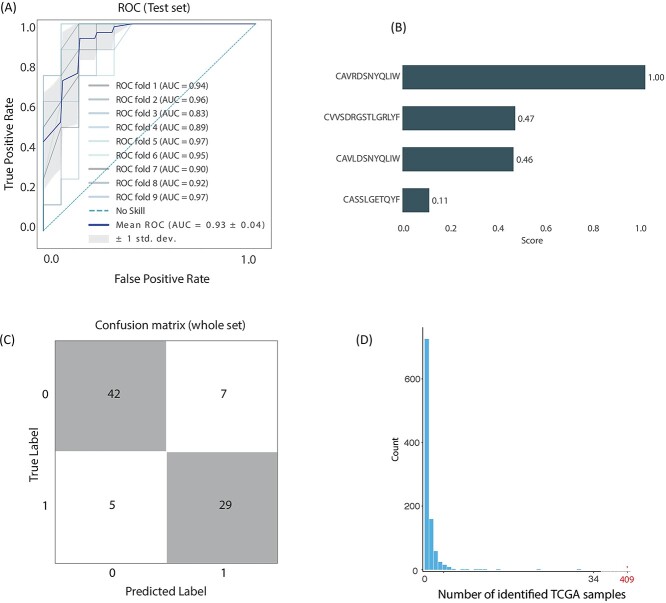
Results of top clones classification and feature selection. (**A**) A receiver operating curve representing multiple data splits shows robustness of the algorithm and an average ROC curve in a darker shade. Average AUC value is 0.93. (**B**) As explained in the text, the final model was able to use four features (four clones) to achieve optimal results. The CDR3 sequences of these four clones are reported in the panel. Interestingly, the first clone and the third clone belong to a MAIT cell and the second to an iNKT cell. (**C**) A confusion matrix showing performance over entire dataset. (**D**) To test the significance of the four clones presented in (B), we repeatedly, randomly, selected four TCRs and searched for them in the TRUST4 TCGA dataset. This was repeated 1000 times, with the number of appearances of at least one of the four TCRs recorded. As the panel indicates, the four TCRs we selected for the LDA model appear in significantly larger frequency.

Results (Figure 3) show that the features reached optimal performance when used in an XGB model with a feature set of size 8 selected using the LDA estimator for the backward-SFS algorithm. Results gave an AUC of 0.93 on the test set. Again, as with the top–down methodology, we used feature ranking to show that not all eight features in the set are required to achieve a highly accurate model. Therefore, we performed backward-SFS to reduce the number of features from 16 to 4 and then retrained all models. Under these settings, an LDA model, with only four features, gave an average AUC of 0.93 (Figure 3A) on multiple splits of the data. Feature ranking is shown in Figure 3B. Details on the specific (BO selected) hyperparameters of the model are included in the DOME description (Supplementary Table 2) and in Methods. Figure 3C illustrates the classification outcomes for the entire dataset (train + test).

### Identification of significant TCRs

Specific T-cell subtypes are identifiable through the V(D)J combination that is responsible for their TCR sequences [26]. The top–down methodology describes above provided us with three TCRs: CVVSDRGSTLGRLYF, CASSLKETQYF and CASSLAYEQYF. We identified the highest rank feature, CVVSDRGSTLGRLYF, to be the product of V10 and J18 and therefore an iNKT cell.

The top clones methodology provided us with four features: CAVRDSNYQLIW, CVVSDRGSTLGRLYF, CAVLDSNYQLIW and CASSLGETQYF. The highest rank feature and the third ranked feature, CAVRDSNYQLIW and CAVLDSNYQLIW, respectively, both are product of V1-2 and J33 and are therefore MAIT cells. The second ranked feature, CVVSDRGSTLGRLYF, which is also the highest ranked feature according to the top–down model, is an iNKT cell (as mentioned above).

### Analysis of signified clones

Further investigation of the set of clones produced by the top–down methodology, shows that in VDJdb [20] the highest-ranked TCR (which we have shown earlier to be an iNKT cell) and the third-ranked TCR both have one entry in the database. The second most important TCR has none.

Clearly, due to the methodology, TCRs selected using the top clones approach show an omnipresent behavior. VDJdb shows the most important clone in 16 unique entries, the third in 13 unique entries and the fourth in 5 unique entries (Supplementary Table 3). The second was found in 1 unique entry.

We looked at the clones in the McPAS database [19]. The top–down TCRs showed 391 entries for the most important clone, 2 entries for the second and no entries for the third. The top clones showed 279 entries for the most important clone, 391 for the second, 122 for the third and 3 for the fourth (Supplementary Table 4).

### Occurrence of clones in tumor tissue

Tumor tissue is often rich with tumor-infiltrating lymphocytes (TILs), and recent work [21] provides a large dataset which contains TILs from The Cancer Genome Atlas (TCGA). This dataset offers an opportunity to search for the clones that proved to be important features in our classification task. Simply counting appearances of these TCRS showed that the three TCRs identified by the top–down methodology appeared in 82 samples (a sample is counted if one of the three TCRs was identified in it). The four TCRs from the top clones set appeared in 409 samples. To associate significance with occurrence in the set's appearance in tumor samples (82 samples, 409 samples), we performed bootstrapping by randomly selecting three (or four) TCRs from the full set of TCRs in our dataset and performing the same count with these selected sequences. That is, we searched for this random set in the TRUST4 TCGA dataset and counted the number of sequence appearances in the samples. We repeated this process 1000 times to produce the distribution shown in Figure 2D. As the figure shows, the event of 82-samples is significantly far from the produced distribution, with *P*-value < 10^−3^. In a similar manner, we produced the distribution in Figure 3D for the four top clones TCRs we identified. As the figure shows, the event of 409-samples is significantly far from the produced distribution, with *P*-value < 10^−3^.

**Figure 4:**
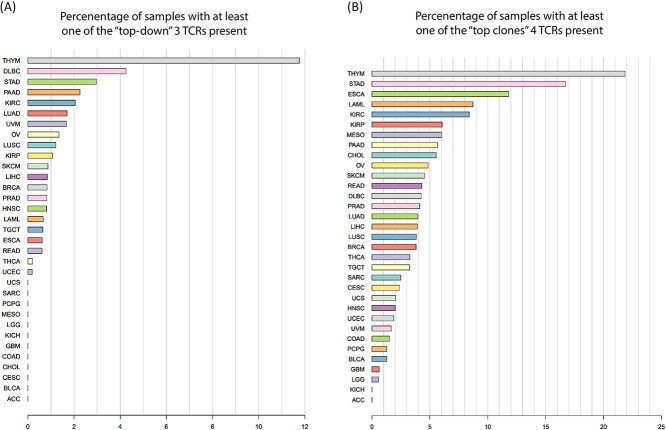
Distribution of samples containing at least one of the most important TCRs for our model across different cancer types. Samples are from the TCGA TCR dataset. Each bar corresponds to a specific cancer type and illustrates the proportion of samples in which at least one of the TCRs was detected. The colors of the bars in the graph are for visual distinction only and do not represent any specific meaning. (**A**) Represents the distribution of the three TCRs chosen with the top–down method. (**B**) Represents the distribution of the four TCRs chosen with the top-clone Method. STAD, stomach adenocarcinoma; ESCA, esophageal carcinoma; LAML, acute myeloid leukemia; THYM, thymoma; KIRC, kidney renal clear cell carcinoma; KIRP, kidney renal papillary cell carcinoma; PRAD, prostate adenocarcinoma; OV, ovarian serous cystadenocarcinoma; LIHC, liver hepatocellular carcinoma; BRCA, breast-invasive carcinoma; DLBC, lymphoid neoplasm diffuse large B-cell lymphoma; LUAD, lung adenocarcinoma; TGCT, testicular germ cell tumors; THCA, thyroid carcinoma; PAAD, pancreatic adenocarcinoma; HNSC, head and neck squamous cell carcinoma; SARC, sarcoma; READ, rectum adenocarcinoma; LUSC, lung squamous cell carcinoma; MESO, mesothelioma; SKCM, mesothelioma; COAD, colon adenocarcinoma; UCEC, uterine corpus endometrial carcinoma; PCPG, pheochromocytoma and paraganglioma; GBM, glioblastoma multiforme; CESC, cervical squamous cell carcinoma and endocervical adenocarcinoma; UVM, uveal melanoma; UCS, uterine carcinosarcoma; LGG, brain lower grade glioma; KICH, kidney chromophobe; BLCA, bladder urothelial carcinoma; ACC, adrenocortical carcinoma.

The identification of these samples (both the 82 and the 409) showed that these TCRs are TILs in multiple tumor types. Figure 4A (for the top–down) and Figure 4B (for the top clones) illustrate for each cancer type the percentage of samples that have at least one of the TCRs present. Interestingly, the set distribution does not signify OC (Supplementary Table 5, Supplementary Table 6).

## DISCUSSION

Oncology refers to ‘liquid biopsy’ as the utilization of relatively-easy-to-obtain biological fluids for cancer decision-making. Various approaches, such as following circulating tumor DNA (ctDNA), circulating tumor cells (CTCs), cell-free RNA (cfRNA) and exosomes [27, 28], aim to facilitate the early detection of cancer or mange treatment.

Efforts are made to monitoring OC for diagnostic and prognostic purposes using ctDNA [29–31]. In many OC studies, the performance of ctDNA platforms is being evaluated in comparison to the levels of the standard serum marker, CA-125, which has played a chief role in screening, detecting and managing OC over the past four decades [32]. However, the CA125 biomarker is not very sensitive during early stages of OC and an elevated serum level of CA125 may be observed in other physiological or pathological conditions [32–34]. For ctDNA, inherent characteristics such as its length, copy number variability and methylation present challenges for detection [35].

To address these limitations in ctDNA-based biomarkers, research now combines ctDNA, with various ‘omics’ such as proteomics, epigenomics, RNA, nucleosomes, exosomes and immune markers [36, 37].

Cohen *et al*. [38] developed the CancerSEEK test, which is an example for overcoming multiple limitations associated with circulating tumor-based material. This study reported that ctDNA was detected in OC with a sensitivity of 98% and specificity of 99%. However, early-stage detection rate was only 38%. The possibility of improving over these results led us to investigate the use of the peripheral T-cell repertoire for early detection.

### Peripheral blood TCR repertoire as a novel approach for early cancer detection

This research explores the potential association between TCR repertoire in peripheral blood and the clinical status of samples categorized as OC patients or as HD. As the results show, the implementation of supervised machine learning techniques proved instrumental in effectively discerning between OC and HD samples, overcoming the limitations of conventional TCR repertoire analysis methods. We approached features analyses from two routes. One route was the naïve top–down elimination of features, using multiple reduction methods, which started with the whole set of features (TCRs) discovered in our repertoire sequencing. The second route has focused on the use of ubiquitous TCRs, which we mined from three datasets: VDJdb, MCPaS and the TRUST4 TCGA study. From these three datasets, we selected the most public clones and used those for our machine learning. Both routes led to excellent performance. The robustness of our approach was evident as it successfully classified new, previously unseen samples, validating the generalizability and practical utility of the model.

### Insights gained from TCR repertoire profiling

Investigation of the key features of the first set (top–down) of TCRs revealed the highest-ranked clone, CVVSDRGSTLGRLYF, to be an iNKT cell, perhaps calling attention to the potential significance of this T-cell subtype in the context of our study. Analyzing this sequence in McPAS [19] further emphasizes the uniqueness of this clone. Interestingly, while this clone is highly prevalent in McPAS, it is relatively rare in VDJdb [20]. The top–down approach led to a total of three TCRs needed for the task of classification. The two other (excepting the highest-ranked CVVSDRGSTLGRLYF) TCRs are rare in both McPAS and VDJdb.

As mentioned above, our second route (termed top clones in Methods) has been to first identify a set of ubiquitous, significant TCRs in the databases (VDJdb, McPAS, TCGA). To do so, we selected the 10 most prevalent TCRs from each database. Due to overlap between the three databases, these 30 TCRs were reduced to 25 unique TCRs. Among these 25, 16 TCRs were found in our produced dataset (the dataset we generated by repertoire sequencing the HD and OC blood samples). Finally, through the methodical process of feature selection described in Methods, we reduced the set of 16 to 4 TCRs (four features) that are used in the model that performs the classification.

Notably, within the VDJdb dataset, 8 out of the 10 selected TCRs were present in our dataset. One of the four final TCRs, CAVRDSNYQLIW, originated from this subset of 8. This TCR had 16 unique entries in VDJdb. Two additional TCRs out of the four were not in the top 10 of VDJdb but still well represented with 13 and 5 entries. Remarkably, these entries surpassed 99.5% of all TCRs within the VDJdb in terms of frequency.

Similarly, the McPAS dataset contributed significantly, with 8 out of the 10 selected TCRs present in our dataset. Three out of the four final TCRs originated from McPAS.

In the TRUST4 TCGA dataset, 5 of the 10 selected TCRs were identified in our dataset, and 2 of these 5 were among the final set of four chosen TCRs. The remaining two TCRs, although not ranked within the top 10, demonstrated significant representation with 115 and 32 entries, surpassing 99.97% of all TCR sequences in the database in terms of frequency.

The investigation of these clones in the TRUST4 TCGA dataset further shows interesting patterns. Notably, the presence of the four TCRs is widespread across various tumor types. This observation suggests that the identified TCRs may play a role in multiple cancer types. The bootstrapping analysis presented in the Results section demonstrates the non-arbitrary nature of both sets of identified clones (sequences), emphasizing their significance in the context of tumor samples.

The findings also show that although the clones were found in association with the OC signal, they do not seem to be limited to TILs, as they are also found in HD samples.

### Limitations and future directions

This study presents a potential association between TCR repertoire in peripheral blood and patients’ clinical status. Still, several limitations warrant consideration. First, while the limited sample size cannot fully represent the diverse spectrum of clinical conditions within OC, thus limiting the generalizability of our findings to broader populations, it is imperative to emphasize the heterogeneous nature of our dataset. Notably, in its present form, the data may not reliably discriminate between TCR repertoires of different OC histotypes. Further, variability in sample sizes required subsampling. Therefore, the subset chosen may not fully represent the entire dataset, which may influence the robustness of the results. Finally, the TCRs identified, while informative, may not necessarily be specific to OC, potentially involved with various cancer types or even various pathologies. Addressing these limitations through larger, more diverse cohorts will be a required next step in moving forward with these concepts.

## CONCLUSIONS

In this study, we showed that OC tumors associate with differences in the TCR repertoire in peripheral blood. The repertoire segment of peripheral blood is information-rich. This information comes from the minute differences between any two TCRs. When scaled to the order of millions of T cells, these differences provide large amounts of data that are different between any two individuals. The challenge is transforming these seemingly random data into disease-relevant information [39]. Our findings here support the proposition that tumor-relevant signal is maintained by the immune system and is coded in the T-cell repertoire. The TCR repertoire has demonstrated a capacity for picking up this signal [40–42]. It seems as if changes made by the tumor lead to a response in the immune system. If this response happens early enough, and if we are able to pick up on this response using repertoire technologies, then this might be a pragmatic way to search for early signs of cancer, with minimal patient burden, possibly with enhanced sensitivity. To substantiate these claims, more samples are needed with specific information about tumor stages. Such future information would inform us of the association between the signature and the potential for early detection.

**Key Points**

- We quantified the immunological repertoire of ovarian cancer patients and healthy donors and mined these data with computational tools.
- Using machine learning, we developed a model that can stratify ovarian cancer patients and healthy donors.
- The model is composed of four intriguing T-cell receptors.
- We have shown clear evidence for an association between the existence of a tumor and information from peripheral blood.

## Supplementary Material

SuppFig01_bbae075

Supp_Table_1_bbae075

Supp_Table_2_bbae075

Supp_Table_3_bbae075

Supp_Table_4_bbae075

Supp_Table_5_bbae075

Supp_Table_6_bbae075

## Data Availability

The data that support the findings of this study are available on Zenodo. Full details are also included in the added DOME report.

## Author Biographies

**Sarit Aviel-Ronen** born on September 22, 1967 in Tel Aviv, Israel. She received BS degree (1988–1992) and MD (1992–1996) in medicine from Technion, Israel Institute of Technology, Haifa, Israel. She also completed a GCP course in 2009 from Bio-Medical Research Design Ltd, Sheba Medical Center, Tel-Hashomer, Israel and Bio-Medical Informatics Program in 2013–2014 from Bar-Ilan University, Life Sciences, Ramat-Gan, Israel. She worked as an intern in 1996 from Kaplan Medical Center, Rehovot, Israel Sarit Aviel-Ronen; resident of pathology in 1997–2002 from Sheba Medical Center, Tel Hashomer, Israel; Senior pathologist in 2002–2003 from Sheba Medical Center, Tel Hashomer, Israel; post-doctoral research fellow/molecular pathology research fellowship in 2004–2007 from University Health Network – Ontario Cancer Institute/Princess Margaret Hospital & University of Toronto – Faculty of Medicine, Toronto, Canada; 2007–2017: Senior pathologist/Sheba Medical Center, Tel Hashomer, Israel; medical director of Patho-Lab Diagnostics Ltd, Ness Ziona, Israel in 2017–2020; senior pathologist & researcher for Agilent Technologies, Research Laboratories, Petah Tikva, Israel in 2021–2022; senior lecturer of head of pathology course for medical students/head of translational research in molecular pathology laboratory in 2020-present at Adelson School of Medicine, Ariel University, Israel; senior pathologist at Pathology Department, Sheba Medical Center, Tel-Hashomer, Israel and consulting pathologist for Agilent Technologies, Research Laboratories, Petah Tikva, Israel in 2022-present.

Positions in academic administration: From 2020 to present, she was the member of the teaching committee of the Adelson School of Medicine, Ariel University, Israel; member of the research committee of the Adelson School of Medicine, Ariel University, Israel in 2022-present; editor or member of editorial board of scientific or professional journal; member of editorial board – Scientific Reports Membership in professional/scientific societies in 2014-present; the Association for Molecular Pathology (AMP, 2023-present); the International Society of Gynecological Pathologists (ISGyP, 2021-present); the Israeli Society of Gynecologic Oncology (ISGO) representative in the Gynecological Cancer InterGroup (GCIG, 2018-present) Pathology Liaison Group and the Committee for Rare Tumors; Israel Medical Association (1997-present); European Society of Pathology and a member of the Gynecological Pathology working group since 2017 (2014-present) and in 2005–2007: American Association of Cancer Research.

Educational activities: instructor in pathology course of Department of Pathology, Sackler School of Medicine, Tel-Aviv University, Israel in 1997–2003; 1998–2003: lecturing in pathology courses, School of Dental Medicine, Academic School of Nursing at the Sheba Medical Center, Department of Communication Disorders, Speech, Language and Hearing. School of Health Professions, Sackler School of Medicine, Tel-Aviv University, Israel; 2001–2003: lecturing in general pathology course for medical students, Department of Pathology, Sackler School of Medicine, Tel-Aviv University, Israel; 2004–2007: post-doctoral research fellowship at the Ontario Cancer Institute/Princess Margaret Hospital, Toronto, Canada; 2007–2019: lecturing in pathology courses for medical students, Department of Pathology, New York program and Dr. Miriam and Sheldon G. Adelson Graduate School of Medicine, Sackler School of Medicine, Tel-Aviv University, Israel; 2013–2019: coordinator of “The Reproductive System” course for medical students, jointly, Department of Pathology, Sackler School of Medicine, Tel-Aviv University, Israel; 2019-present: heading and lecturing in pathology course in the Department of Occupational Therapy, School of Health Sciences, Ariel University, Israel; 2020-present: senior lecturer in the Adelson School of Medicine, Ariel University, Israel, Heading and lecturing in pathology course for medical students.

(a) Honors, citations, awards (including during studies): 1989: Technion, Israel Institute of Technology, Haifa, Israel – President's award; 1990–1992: Faculty of Medicine, Technion, Israel Institute of Technology, Haifa, Israel – Dean's award; 1997: Faculty of Medicine, Technion, Israel Institute of Technology, Haifa, Israel – Dean's award for M.D thesis; 2006: The Pulmonary Pathology Society's Trainee Presentation Award – for poster presentation at the USCAP Annual Meeting 2006 in Atlanta, Georgia, USA; 2012: Sackler School of Medicine, Tel-Aviv University, Israel – Dean's award for outstanding lecturer of Department of Pathology.

(b) Fellowships: 2004–2005: CIHR (Canadian Institute of Health Research) – $60,000 per year – Molecular Oncologic Pathology Research Fellowship at University Health Network – Ontario Cancer Institute/Princess Margaret Hospital, Toronto, Canada; 2006–2007: OCI (Ontario Cancer Institute) – $60,000 per year – OCI Knudson Research Fellowship for molecular oncologic pathology; 2006–2007: NCIC (National Cancer Institute of Canada) – $40,000 per year – Terry Fox Foundation Clinical Research Fellowship for 2 years. The research project: Signature genes for predisposition to brain metastasis in non-small cell lung cancer (NSCLC).
