# Supplementary figures and images for: Ovarian cancer is detectable from peripheral blood using machine learning over T-cell receptor repertoires
Source: Brief Bioinform. 2024 Mar 13;25(2):bbae075. doi: 10.1093/bib/bbae075 (PMC10938541; doi:10.1093/bib/bbae075)

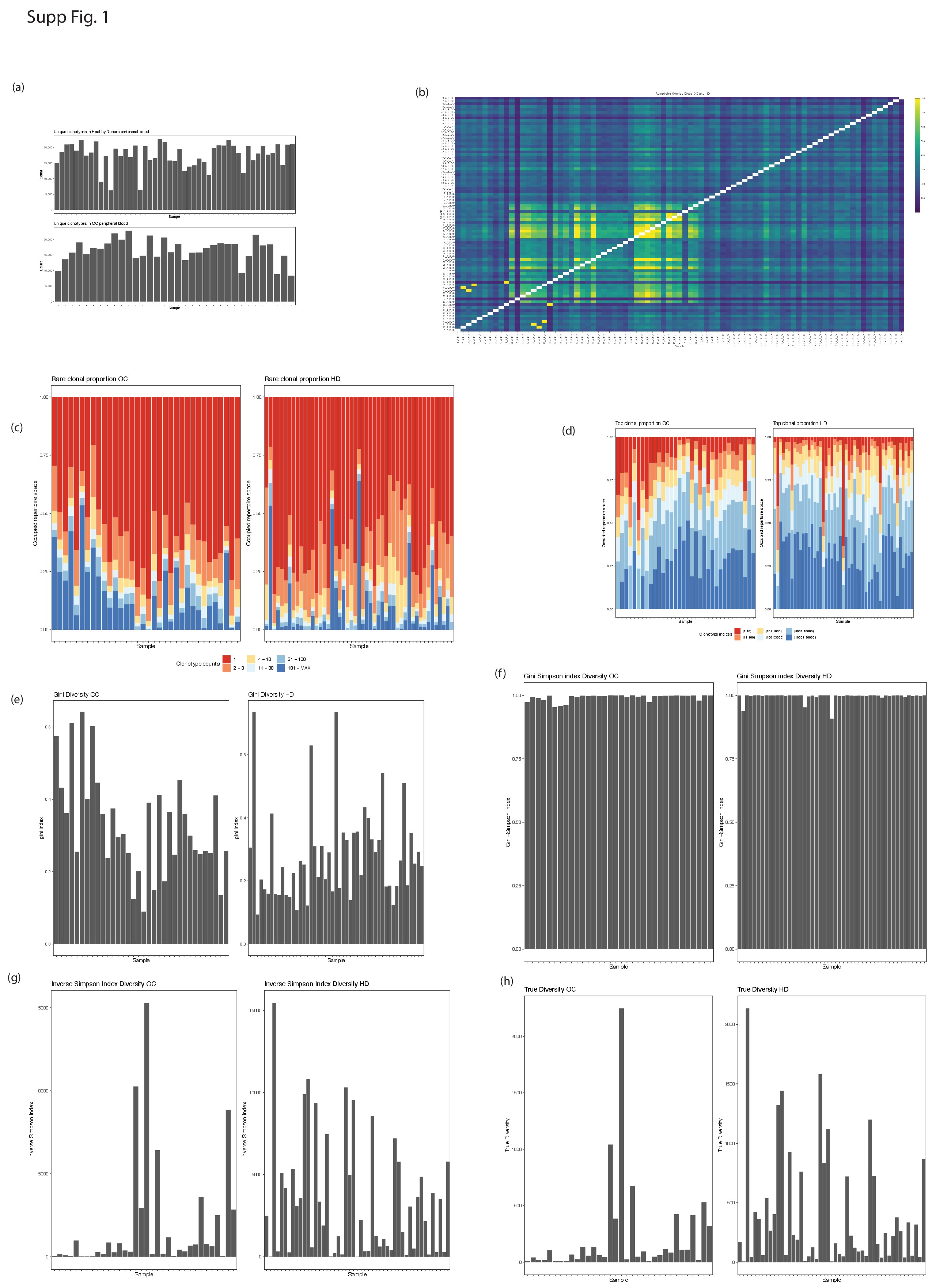

Supplement: SuppFig01_bbae075 [file suppfig01_bbae075.jpeg]
